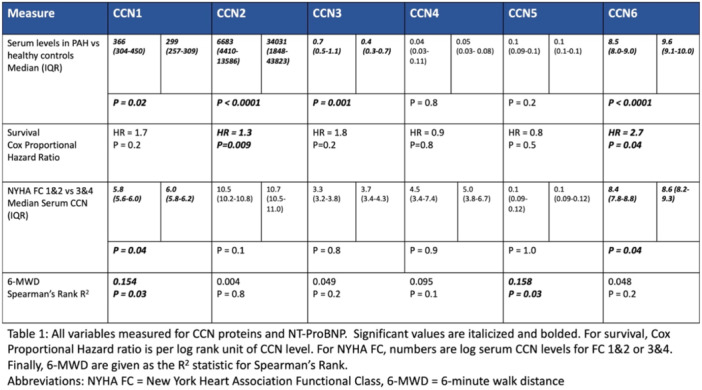# Abstracts from the 17th International Neonatal and Childhood Pulmonary Vascular Conference

**DOI:** 10.1002/pul2.12369

**Published:** 2024-04-24

## A001 BETTER LATE THAN NEVER: A RARE LATE PRESENTATION WITH CAREFUL CONSIDERATION OF SURGICAL CANDIDACY

Wagner JD, Ligon RA, Shashidharan S, Wilson HC, Kanaan UB

Emory University School of Medicine, Atlanta, Georgia; Children's Healthcare of Atlanta, Atlanta, Georgia, USA

Background: A 7‐year‐old male Sudanese immigrant was referred to the cardiology clinic for a history of unknown congenital heart disease. Symptoms included tachypnea at rest and poor growth. Vital signs showed SpO2 of 94% and a BMI in the 2nd percentile for age. Physical exam was notable for a hyperactive precordium, bounding pulses, an ejection click, single S2, 4/6 harsh systolic ejection murmur, and 2/4 decrescendo diastolic murmur. Case Materials: Echocardiogram showed truncus arteriosus type 1 with unobstructed branch pulmonary arteries arising from a short main pulmonary artery segment. There was low normal ventricular systolic function and a tri‐leaflet truncal valve with mild stenosis and mild regurgitation. Cardiovascular magnetic resonance imaging (CMR) and cardiac catheterization were obtained to assess surgical candidacy. CMR showed normal biventricular systolic function, moderate‐severe biventricular dilation, mild truncal insufficiency, and a Qp:Qs of 3.5:1. Cardiac catheterization showed a Qp:Qs of 4.3:1 and PVRi of 2.3 WU*m^2^ during baseline conditions of 21% FiO2 with reduction in PVRi to 1.5 WU*m^2^ with 100% FiO2. Case Result: Though the clinical presentation suggested a significant shunt and low PVR, given the high risk of pulmonary vascular disease in a 7‐year‐old with truncus arteriosus, a complete investigation was performed using a multi‐modal approach to decide surgical candidacy. CMR and catheterization confirmed the clinical and echocardiographic impression. He subsequently underwent truncus arteriosus repair using a 20 mm RV‐to‐PA homograft conduit and the creation of a 3 mm atrial septal defect. Postoperative echocardiogram showed a bidirectional atrial level shunt and ½ systemic right ventricular pressure, which improved after initiation of a pulmonary vasodilator. He otherwise had an uncomplicated post‐op course and was discharged on post‐op day 9. Conclusion: Patients with unoperated truncus arteriosus with unguarded pulmonary arteries have a high risk for pulmonary vascular disease. Successful late repair is possible, but careful assessment before surgery is imperative.

## A002 CASE PRESENTATION: LATE PRETERM INFANT WITH SEVERE PULMONARY HYPERTENSION AND BRONCHOPULMONARY DYSPLASIA

Sabourin, L, Riker ME, Jackson EO, Radman M, Yung D

Seattle Children's Hospital and University of Washington, Seattle, Washington, USA

A 5‐week‐old, ex 34‐week infant with IUGR transferred with bronchopulmonary dysplasia (BPD), poor growth, worsening respiratory failure, and severe pulmonary hypertension (PH) by echocardiogram. She was on CPAP with 40% oxygen, and had dysmorphic features, high arched palate, and polydactyly. Initial echocardiogram showed suprasystemic RV pressures with right to left shunt through a small ventricular septal defect, bidirectional shunt through a small atrial septal defect, dilated and hypertrophied RV with moderately diminished function. Imaging included chest X‐ray with 13 ribs, hazy bilateral lung opacities, and streaky consolidation; chest CTA showed severe lung disease with diffuse patchy and ground glass opacities throughout lung fields. Lung disease seemed more severe than expected for gestational age, so the working diagnosis was developmental lung disease (DLD). She was intubated and muscle relaxed. Milrinone caused hypotension and hypoxia. There was no response to iNO. Post‐pyloric feeds and diuretics were initiated. Rapid whole exome sequencing showed 16p11.2 deletion. A lung biopsy was not performed due to instability. Three high‐dose pulse steroid courses were administered. Each led to improvement in ventilation and pulmonary hypertension by echocardiogram. At 3 months of age, she underwent a lung biopsy. Findings showed severe BPD, mild pulmonary interstitial glycogenosis (PIG), and severe pulmonary arterial vasculopathy. She was treated with a BPD ventilator strategy and low‐dose prednisolone. At 4 months of age, she was extubated. At 5 months of age, cardiac catheterization showed mean pulmonary artery pressure 32 mmHg and Qp:Qs 1.4. The ASD was device closed. At 7 months of age, she was on 6 L HFNC and 21% oxygen. Echocardiogram showed no evidence of PH. The severe clinical presentation led to suspicion for DLD, but lung biopsy and genetics showed only BPD. Continued BPD‐directed treatment led to improvement and eventual PH resolution. The responsiveness to high‐dose steroids may be explained by PIG.

Lung biopsy slides


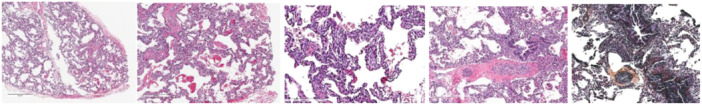


## A003 RACIAL DISPARITIES IN URINE AMPHETAMINE TESTING AMONG PATIENTS SEEN IN UCSF PULMONARY HYPERTENSION CLINIC

Emily Sumpena, BA, Marc A. Simon, MD, MS, Joseph Bayne, MD, Jacqueline T. DesJardin, MD

University of California, San Francisco, California, USA

Background/Hypothesis: Amphetamine use is increasingly common and implicated in the pathogenesis of pulmonary arterial hypertension (PAH). Patients with methamphetamine‐PAH have worse outcomes than other PAH etiologies. Active methamphetamine use is often considered a contraindication for advanced therapies, such as intravenous prostacyclins or lung transplantation. Thus, the best practice would be to screen all patients with PAH for stimulant use. However, the decision of whether to screen is currently made on an individual basis by providers, who may be influenced by implicit biases. The purpose of this study is to investigate if there are racial disparities in urine toxicology screening among patients seen in the UCSF Pulmonary Hypertension (PH) Clinic. Materials and Methods: Patients seen in the UCSF PH Clinic between February 2021 and March 2023 were identified using the UCSF De‐identified Clinical Data Warehouse (DeID CDW). Toxicology screening orders and results were extracted from the electronic medical record. Patients who received toxicology screening at UCSF were compared to those who did not. Screening practices and testing positivity were examined by race. Results: Only 238 of 625 patients (38%) seen in the UCSF PH Clinic had urine toxicology testing performed in the UCSF Health system. The majority of providers who ordered urine toxicology screening were cardiologists (29%), general medicine physicians (27%), and pulmonologists (14%). Patients who received toxicology screening were more likely to be prescribed pulmonary vasodilators. Among those tested, 23% (*n* = 54) tested positive for stimulants at least once. Latinx patients were most likely to be screened of any racial groups (48% Latinx, 38% Black, 39% White, and 33% Asian). However, white patients were most likely to test positive when screened (34% White, 17% Black, 12% Latinx, and 4% Asian) (Figure 1).


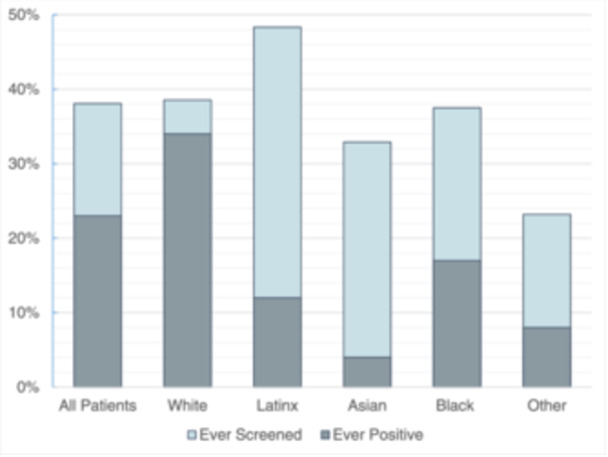


**FIGURE 1** Toxicology screening and test positivity rate by race in PH clinic patients.

Conclusions: Despite endemic amphetamine use in California, a minority of patients seen in UCSF PH Clinic are screened for amphetamine use. PH cardiologists and pulmonologists are often the providers sending toxicology testing, and they need to be trained and educated in discussing this sensitive testing with patients. While screening outside UCSF was not captured, underscreening likely still exists. Latinx patients seem to be over‐screened for amphetamine use, while white patients are under‐screened. Because testing positive for amphetamines has implications on candidacy for advanced PAH therapies, it is important to implement universal screening for all patients with PH to reduce potential racial biases and disparities in treatment. Future Directions: Since September 2023, UCSF PH Clinic has formalized a standard protocol for urine toxicology screening, which will hopefully reduce disparities in toxicology screening. Providers will obtain random urine toxicology on all patients when they initiate care at UCSF, or are being considered for parenteral or advanced therapies. Additional research will be required to determine if such standardized protocols are effective in reducing racial disparities in toxicology screening.

## A004 AORTOPULMONARY COLLATERALS IN SEVERE PEDIATRIC PULMONARY ARTERIAL HYPERTENSION

Gray JM, Bhalla S, Bugenhagen SM, Grady RM, Drussa A, Eghtesady P, Aggarwal M

Division of Pediatric Cardiology and the Mallinckrodt Institute of Radiology, Washington University in St. Louis, St. Louis, Missouri, USA

Background & Hypothesis: Pediatric pulmonary arterial hypertension (PAH) is a severe, progressive disease with multiple etiologies. Pediatric and adult pulmonary hypertension may have different physiology with differing degrees of right ventricular pressure estimation. Computed tomography angiography (CTA) is used to evaluate PH with limited reports in pediatrics. Here we evaluate previously described imaging features in pediatric patients. Materials and Methods: Nineteen children with severe pulmonary hypertension with CTAs evaluated at a single institution between January 2017 and December 2023 were retrospectively reviewed. Patient data including age, genetic diagnosis, details of PAH treatment, and CTA findings were assessed. The presence and burden of aortopulmonary collaterals were gauged on a scale of one to four. Frequency analyses of the above findings were calculated, and comparative analyses performed between those with and without known genetic mutations utilizing Fisher's exact test. Results: In these 19 patients, 7 (33%) were female with the following etiologies: 8 genetic, 5 idiopathic and 3 congenital heart disease, and 3 with other types. All patients had suprasytemic PH and were on three classes of pulmonary vasodilators. Median length of treatment was 4.3 years. All patients had APCs identified with 2 with Type 1 (11%), 8 with Type 2 (42%), 7 with Type 3 (37%), and 2 with Type 4 (11%). Neovascularity was present in 18 subjects (95%). There were no differences in APC score between Genetic PAH versus other causes (2.3 vs 2.6). Other CTA findings are shown in Table 1. No patients had episodes of hemoptysis. Conclusions: This is the first demonstration of APC formation in children with severe PAH. The presence of APCs is reported to exclude PAH in adults. Impetus for formation and importance of these vascular changes to long‐term outcomes, hemoptysis, and potential surgical interventions (reverse Potts shunt and transplantation) remains to be determined.

**Table:** 

Patient data	*N* = 19 (%)
Female patients	7 (33%)
Age (median–IQR)	11.4 (6.1–16.3)
Type of PH	
Genetic	8 (38%)
IPAH	5 (24%)
CHD	5 (24%)
Prematurity	1 (5%)
Mixed/other	2 (9%)
Findings	
Aortopulmonary collaterals	19 (91%)
Neovascularity	18 (88%)
Perivascular halos	14 (67%)
Mosaic attenuation	5 (24%)
Lymphadenopathy	1 (5%)
Bronchiectasis	0 (0%)

## A005 OUTCOMES OF TRANSCATHETER ATRIAL SEPTAL DEFECT CLOSURE IN SMALL INFANTS WITH DEVELOPMENTAL LUNG DISEASE

Jackson EO, Carlozzi L, Riker M, Sabourin L, Cody H, Choi C, Johnston TA, Rubio AE, Morray MH, Handley S, Eldredge L, Berkelhamer S, Yung, D

Seattle Children's Hospital and University of Washington, Seattle, Washington, USA

Background/Hypothesis: Pulmonary hypertension (PH) associated with chronic lung disease is categorized as Group 3 PH by the World Symposium on Pulmonary Hypertension classification system. Neonatal chronic lung diseases are increasing in prevalence, and there are insufficient therapeutic options for PH in these cases. Patients with developmental lung disease (DLD), including bronchopulmonary dysplasia (BPD) and genetic syndromes, are at risk for developing PH. Closing atrial septal defects (ASD) has been reported to improve BPD PH. We hypothesized that ASD closure in small infants (<6 kg) is feasible and may result in improvement in respiratory support and PH. Materials and Methods: We reviewed records of hospitalized infants born between 2020 and 2023, with transcatheter ASD closure performed at a weight less than 6 kg. We collected data on demographics, PH, and respiratory support, before and after ASD closure. PH by echocardiogram was defined as tricuspid regurgitation (TR) jet ≥ 2.5 m/s, or VSD jet predicting RV pressure ≥ 25 mmHg. Before ASD closure, RV dilation and septal flattening without measurable TR jet was defined as “unclear” due to volume overload. After ASD closure, septal flattening was considered a PH finding. Respiratory support was tracked only through hospital discharge. Results: There were 16 patients with no procedural complications. 67% of patients had BPD and 25% had Trisomy 21 (Table 1). At ASD closure, the median post‐menstrual age (PMA) was 45.1 weeks, and median weight was 3.9 kg. At the time of procedure, three patients required mechanical ventilation, 4 on CPAP, and 9 on HFNC. At ASD closure, 11 patients had PH, 3 were unclear, and 2 had no PH. At 3 months after procedure, two patients remained on mechanical ventilation, and all other patients were on LFNC or RA. At 6 months after procedure, no patients had PH (Figures 1 and 2). Conclusions: Transcatheter ASD closure is safe in small infants with developmental lung disease and may contribute to the weaning of respiratory support and resolution of PH. Randomized studies to evaluate this intervention in DLD are greatly in need.

TABLE 1 Demographics and patient characteristics.

**Table:** 

	*N* = 16 Median (IQR)
Female, *n* (%)	12 (67%)
Birth weight, kg	0.91 (0.6–1.23)
Gestational age, weeks	28.2 (26.1–34.7)
≥37 weeks	1
34–36 weeks	4
32–34 weeks	0
28–32 weeks	3
<28 weeks	8
Diagnosis	
Bronchopulmonary dysplasia	12 (67%)
Congenital diaphragmatic hernia	1 (6%)
Trisomy 21	4 (25%)
Weight at procedure, kg	3.9 (3.5–4.13)
Age at procedure, PMA	45.1 (43.2–48)
Discharge date from procedure, days	49 (29–87)


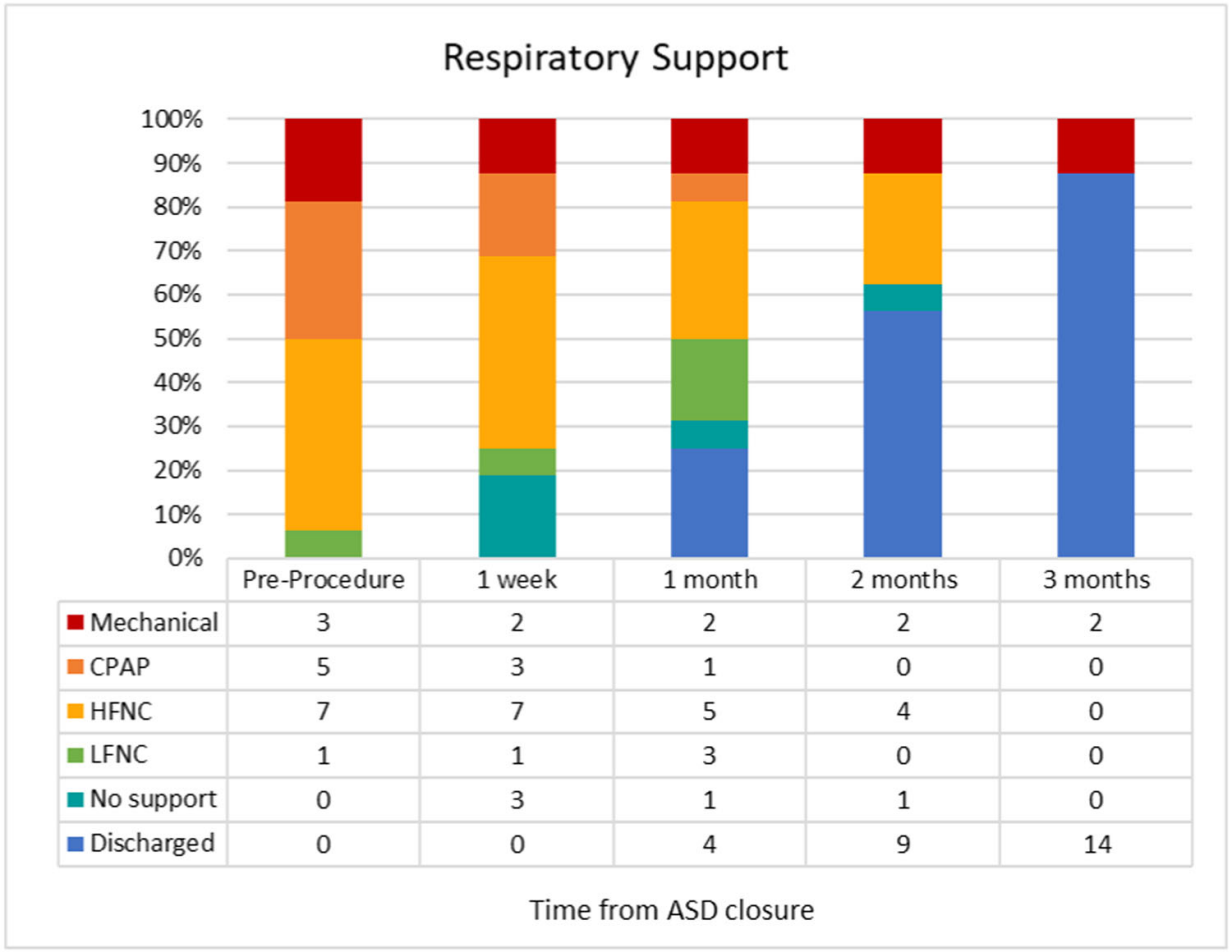


**FIGURE 1** Change in respiratory support.


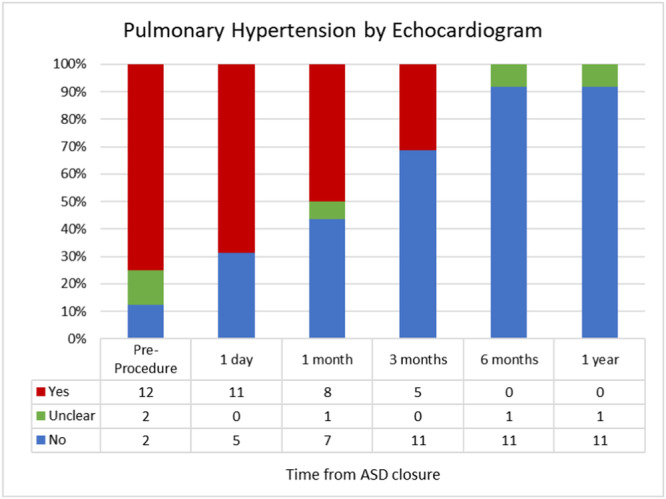


**FIGURE 2** Resolution of PH.

## A006 ANXIETY AND DEPRESSION SCREENING OF YOUTH IN PEDIATRIC PULMONARY HYPERTENSION CLINIC: A MULTI‐CENTER, CROSS‐SECTIONAL STUDY

Parker, C., Whalen, E., Becerra, J., Stevens, L., Smith, MA., Avitabile, CM., Brown, A., Cash, M., Jackson, E., McSweeney, J., Miller‐Reed, K., Reyes, J., Sheppard, C., Mullen, M.P.

University of California, San Francisco, San Francisco, California, USA; Texas Children's Hospital, Houston, Texas, USA; Children's Hospital of Philadelphia, University of Pennsylvania, Philadelphia, USA; Vanderbilt University, Nashville Tennessee, USA; Cinncinati Children's Hospital, Cinnicinati, Ohio, USA; Seattle Children's Hospital, University of Washington, Seattle, Washington, USA; Boston Children's Hospital, Harvard University, Boston, Massachusetts, USA; University of Colorado, Aurora, Colorado, USA; The Hospital for Sick Children, University of Toronto, Ontario, Canada, USA; Stollery Children's Hospital, Alberta, Canada, USA.

Background: The prevalence of anxiety and depression (AD) has nearly doubled in recent studies since the COVID‐19 pandemic began (Racine et al., 2021), and children with chronic diseases are thought to hold a higher risk for the development of AD than healthy peers. Pulmonary hypertension (PH) is a disease marked by high morbidity and mortality with the potential to impact normal growth and development patterns. As a chronic disease with an increased risk for AD development, we identified a gap in evidence for the prevalence of AD and AD screening practices for children diagnosed with PH. Materials and Methods: We developed a multi‐center study with 10 PH centers from North America. Patients enrolled were given GAD‐7 and PHQ9(9A) for AD screening and caregivers completed a supplemental survey to obtain SES data and mental health history. Sites performed a retrospective chart review for PH clinical metrics. Patient demographics and clinical characteristics were described using standard descriptive statistics. Odds of AD associated with select predictors were derived from univariate logistic regressions. Results: A total of 88 patients (ages 12–22 years) were enrolled in the study (female = 54). More than half (46; 51.7%) exhibited at least mild symptoms of anxiety and or depression. In all metrics assessed, there was no statistical significance to suggest an increased risk of AD related to PH severity, mental health history, family dynamics, SES status, and or race and ethnicity. Females were more likely to report AD as compared to males (OR 2.67, 95% CI 1.11–6.61, *p* = 0.030). Conclusions: Of those patients screened, greater than 50% of subjects exhibited symptoms of at least mild anxiety and or depression. This rate was found to be higher than healthy peers and consistent with trends in comparative chronic disease populations. Our data suggests there are no predictive models for identifying PH patients with a higher risk for AD, making screening all PH patients of paramount importance to properly identify AD and provide intervention as necessary. The high prevalence of AD in our study suggests routine AD screening should be provided at standard outpatient clinic visits. As a subsequent beneficial outcome of AD screening, 33 of the patients in this study were provided direct mental health education/counseling by SW or another mental health professional, 15 patients were referred for psychotherapy, and 9 patients were further assessed for suicide safety and were referred to psychotherapy. Including AD screening in future pediatric PH care guidelines should be considered.

## A007 PULMONARY VENOUS COLLATERALS IN PEDIATRIC PULMONARY VEIN STENOSIS AND ASSOCIATIONS WITH PULMONARY VASODILATOR THERAPY

Liliam Aquino MD, Elena Amin MBChB

UCSF Benioff Children's Hospital, San Francisco, California, USA

Background: Pulmonary vein stenosis (PVS) is a challenging disease with poor outcomes. The development of collateral vessels may divert pulmonary venous blood from stenotic veins. We aim to describe the frequency of exposure of pulmonary vasodilators in patients with and without pulmonary venous collaterals in pediatric patients with pulmonary vein stenosis and determine whether there is any association with collateral development. Materials and Methods: Single‐center retrospective cohort analysis of all patients <18 years of age with PVS with angiographic studies between 2013 and 2023. Results: Over the 10‐year study period, 57 individual patients with PVS were identified. The median age at time of diagnosis was 153 days (11 days–15 years). For the entire cohort, 82% (47/57) received pulmonary vasodilators (21/57 prostacyclin, 40/57 phosphodiesterase 5 inhibitors, 32/57 endothelin receptor antagonist). Collateral vessels (Group 1) were present in 28 patients (49%) and not identified (Group 2) in 29 (51%) patients. In Group 1, 89% (25/28) of patients were exposed to pulmonary vasodilators vs 76% (22/29) in Group 2. There was no significant difference between the presence of collaterals based on exposure to any pulmonary vasodilators or specific class of pulmonary vasodilator. Patient survival at the last follow‐up was 19/28 (68%) in Group 1 vs 21/29 (72%) in Group 2, with a median follow‐up time of 2.9 years (12 days–11 years). Conclusions: In our pediatric PVS cohort, both pulmonary venous collaterals and exposure to pulmonary hypertension medications are common findings. In this single‐center cohort with a limited sample size, there was no significant association between exposure to pulmonary vasodilators and the development of pulmonary venous collaterals. Multicenter studies are needed to further explore this question and to understand whether there are implications for patient survival.

## A008 PRIOR AUTHORIZATIONS FOR PEDIATRIC PULMONARY HYPERTENSION MEDICATIONS

Merrill KM, Davis A, Jackson EO, Riker M, Yung D

Seattle Children's Hospital and University of Washington, Seattle, Washington, USA

Background/Hypothesis: Pediatric patients with pulmonary hypertension (PH) are treated with expensive PH medications that may not be approved for children. Insurance companies often require prior authorization (PA) to determine coverage. There are few publications about the burden of the PA process, but it is anecdotally time‐consuming and unnecessary. We sought to understand our center's PA burden and eventual outcome, as this may lead to process improvement and clarity for care providers, insurance companies, and families. Materials and Methods: All patients followed by the PH team at Seattle Children's Hospital were queried using Epic, the electronic health record, for PH prescriptions between 2021 and 2023. Specific tools in Epic, such as SlicerDicer and Reports, were used to assist in collecting patient demographics, insurance information, and PA documentation. Each PH medication was counted as one prescription per patient per year, even if there were multiple prescriptions written in that year. Patient demographics, including PH diagnostic group and insurance type, were collected (Table 1). The number of PAs, denials and approvals were recorded by year and medication (Tables 2 and 3). If a medication was denied, but could be obtained in a different formulation (e.g., tablets vs. compound liquid), it was not counted as a “final denial.” There were 283 prescriptions and 215 PAs (76% of prescriptions) in 53 patients (Table 2). Each patient had a mean of 1.8 prescriptions per year. By year, the percentage of PAs per prescription was stable, but the percent of initial and final denials increased from 6% to 12% and 2% to 5%, respectively. Thirteen (4%) of PAs were for compounding. PAs were least commonly required for prescriptions of FDA‐approved sildenafil (27%) and bosentan (44%). PAs are required for all (100%) ambrisentan prescriptions, and more than one PA (143%) is often needed for selexipag, due to multiple tablet strengths. There were only six (3%) final denials: two selexipag and three ambrisentan were covered by patient assistance programs, and one selexipag was ultimately approved after a failed trial of inhaled treprostinil (Table 3). Conclusions: At our center, PAs are an enormous burden, with 76% of all prescriptions requiring PA, yet could be considered unnecessary as 97% were finally approved, and all patients were ultimately able to receive the prescribed medication. The initial denial rate may be increasing over time, and insurance companies may be denying medications that are available through pharmaceutical company patient assistance programs. Further work on types of insurance and pharmacies associated with PA is needed.

TABLE 1 Patient demographics.

**Table:** 

		*N* = 53
Male sex		26 (49%)
Age, years, mean (st dev)		11 (6.2)
Race		
	White	32
	Asian	6
	Black	3
	American Indian	1
	Alaska Native	2
	Other	7
	Multiple	2
Ethnicity	Hispanic	5
Primary Insurance		
	Medicaid	26
	Private	17
	Military	3
	Kaiser	3
	Self‐funded	4
Diagnosis		
	WHO Group 1	45
	WHO Group 3	5
	WHO Group 5 (single ventricle)	3

TABLE 2 Prior authorizations by year.

**Table:** 

Year	Prescriptions (Rx) written	Prior Auth (PA) (% of all Rx)	Initial denial (% of all PA)	Final denial (% of all PA)
2021	76	53 (70%)	3 (6%)	1 (2%)
2022	97	79 (81%)	7 (9%)	1 (1%)
2023	109	83 (76%)	10 (12%)	4 (5%)
Total	283	215 (76%)	20 (9%)	6 (3%)

TABLE 3 Prior authorizations by medication.

**Table:** 

Medication	Prescriptions (Rx) written	Prior Auth (PA) (% of all Rx)	Initial denial (% of all PA)	Final denial (% of all PA)
Amlodipine	10	5 (50%)	1 (10%)	0 (0%)
Sildenafil	48	13 (27%)	1 (8%)	0 (0%)
Tadalafil	89	64 (72%)	4 (6%)	0 (0%)
Bosentan	32	14 (44%)	1 (3%)	0 (0%)
Ambrisentan	27	27 (100%)	3 (11%)	3 (11%)
Macitentan	8	4 (50%)	0 (0%)	0 (0%)
Selexipag	44	57 (130%)	9 (16%)	3 (5%)
Treprostinil	26	13 (50%)	3 (23%)	0 (0%)
Total	283	202 (72%)	22 (11%)	6 (3%)

The number of PAs is different in Tables 2 and 3 due to additional PA being required for compounding and not for specific medications.

## A009 SURVEY OF FOLLOW‐UP CATHETERIZATION USE IN PEDIATRIC PULMONARY HYPERTENSION

Kanaan U, Ivy, D, Berger RMF, Humpl T, Bonnet D, Bowers D, Mariotti Nesurini S, Beghetti M on behalf of the TOPP‐2 Registry

Emory University, Atlanta, Georgia, USA; Children's Hospital Colorado, Aurora, Colorado, USA; Beatrix Children's Hospital, Groningen, the Netherlands; The Hospital for Sick Children University of Toronto, Toronto, Ontario, Canada; M3C‐Necker Hospital for Sick Children, Paris, France; University of Suffolk, Ipswich, United Kingdom; nspm ltd, Meggen, Switzerland; Children's University Hospital and University of Geneva, Geneva, Switzerland

Background/Hypothesis: Initial right heart catheterization (RHC) is the gold standard for diagnosis of pulmonary hypertension (PH). It provides important information, impacts therapy choice, and has prognostic implications. The role of follow‐up RHC (FURHC) is less clear. We hypothesized that there is wide variation in clinical practice regarding FURHC. Materials and Methods: We conducted a survey of pediatric PH centers participating in the Tracking Outcomes and Practice in Pediatric Pulmonary Hypertension‐2 (TOPP‐2) registry regarding their use of FURHC. Results: Thirty of the 31 participating centers responded. Centers had been active for a median of 15–19 years (5–9 years = 4, 10–14 years = 4, 15–19 years = 11, ≥20 years = 11) and actively followed a median of 50–99 patients (<50 = 12, 50–99 = 5, 100–149 = 5, 150–249 = 1, ≥250 = 7). Centers reported a median of 10–49 RHCs (initial and FURHC) per year (<10 = 11, 10–49 = 11, 50–99 = 6, 100–199 = 2). Centers were evenly split (14 yes, 16 no) on the use of routine FURHC in the absence of clinical change. Among centers performing FURHC in the absence of clinical change, the frequency was annually and every 2 years for five centers each; the remaining four centers described a schedule of more frequent assessment soon after diagnosis and increasing intervals over time (with some prioritizing patients on parenteral prostanoids). Most performed FURHC for clinical (27/30) or echocardiographic (23/30) deterioration or unplanned hospitalization due to PH (18/30). Other indications included rising BNP/NTproBNP (11/30) or addition or discontinuation of medication (11/30 and 12/30, respectively). A small minority perform FURHC after clinical improvement (6/30). Conclusions: Practice regarding the use of FURHC in pediatric PH varies widely, even among expert centers. The role of this invasive assessment should be clarified to justify the risks, expense, and hardship that the procedure entails.

## A010 THICK SKIN PROTECTING A HEART UNDER HIGH PRESSURE—A CASE OF SEVERE PULMONARY HYPERTENSION FROM JUVENILE SYSTEMIC SCLEROSIS WITH IMPRESSIVE OUTCOMES

Cheung S, Tsoi S, Parker C, Colglazier E, Amin E, Nawaytou H, Valdovinos RA, Kim S, Fineman J

University of California, San Francisco, California, USA

Background: Juvenile systemic sclerosis (jSSc) is a pediatric autoimmune disease with a prevalence of 3 per million children; only 7% of patients with jSSc will have pulmonary hypertension (PH). However, when present, PH is a leading cause of morbidity and mortality and usually manifests well into the course of established jSSc. Our case is of a 10‐year‐old girl whose initial presentation of positive U3‐RNP antibody jSSc included diffuse skin findings and advanced pulmonary arterial hypertension (PAH), with acute decompensation from a pulmonary hypertensive crisis requiring veno‐arterial extracorporeal membranous oxygenation (VA‐ECMO). Case Presentation: SA is a 10‐year‐old girl with a history of autism and asthma, who presented to urgent care with antalgic gait and dyspnea; the mother reported these changes had progressed over 6 months before presentation. Expedited work‐up revealed cutaneous sclerosis with subsequent decreased flexion and extension, Raynaud's, vasculopathy on capillaroscopy, digital ulceration, arthritis, muscle weakness, failure to thrive, cognitive regression, and PH. Positive U3 RNP antibodies, along with her presentation, led to a diagnosis of jSSc, diffuse cutaneous subtype. Echo on presentation showed systemic right ventricular pressures; CT angiogram showed no evidence of interstitial lung disease. Abdominal ultrasound was absent of any portosystemic shunt. Cardiac catheterization revealed a pulmonary artery pressure (PAP) of 77/41 with a mean PAP of 54 and a CI of 2.4 L/min/m^2^. On baseline conditions (6L NC, FiO2 100%), her PVRi was 18.5 WU, and on condition II (FiO2 100%, iNO 40 ppm), her PVRi was 14.1 WU. She started Mycophenolate Mofetil (Cellcept) and Rituximab for jSSc and Tadalafil, Ambrisentan, and IV Remodulin for PH. Initiation of immunotherapy induced a high fever, resulting in hemodynamic compromise, that included suprasystemic PA pressures and right ventricular failure, requiring intubation, inotropic and vasopressor support, and ultimately VA‐ECMO with atrial septostomy and atrial stent placement. She received pulse‐dose steroids, Obinutuzumab, IVIg, continuous inhaled Iloprost with Remodullin dose uptitration. She was decannulated after 7 days, extubated after 16 days, and discharged after a total length of stay of 67 days on IV Remodulin, Ambrisentan, and Tadalafil for PH treatment and Mycophenolate Mofetil for jSSC treatment. Notably, she was unable to tolerate subcutaneous Remodulin because of her skin thickening. She is now 1.5 years out from her initial presentation, and her PVRi was 1.0 WU on her most recent cardiac catheterization. She is now maintained on Tadalafil and Mycophenoloate Mofetil. Discussion: This case is unique for several reasons: (1) the young age at presentation for jSSc; (2) the initial presentation, which included multiorgan involvement with advanced, life‐threatening SSc‐associated PAH consistent with other case reports of adults with U3 RNP antibody‐positive systemic sclerosis; and (3) the dramatic response to aggressive PH triple therapy, as opposed to its usual refractory PAH course in this setting. This case demonstrates that early recognition and aggressive treatment, including upfront multi‐drug therapy for PAH and ECMO, can dramatically improve outcomes, particularly when treatment for underlying causes (in this case jSSc) is just being initiated.

## A011 TREPROSTINIL IS ASSOCIATED WITH IMPROVEMENT IN RIGHT VENTRICULAR FUNCTION AND DISEASE SEVERITY IN CHILDREN WITH GROUP 3 PULMONARY HYPERTENSION

Parvathaneni, K, Kochanski, J, Zook, N, Liu, E, Arunamata, A, Feinstein JA, Hopper, RK

Division of Cardiology, Department of Pediatrics, Stanford University School of Medicine, Palo Alto, California, USA

Background & Hypothesis: The use of subcutaneous treprostinil (TRE) in children with pulmonary hypertension (PH) secondary to lung disease (Group 3 PH) is not well described. We hypothesized that PH severity and right ventricular (RV) function improve after TRE initiation. Materials and Methods: This was a single‐center retrospective cohort study of children with Group 3 PH treated with TRE between 2006 and 2022. We compared echocardiographic parameters of PH and RV size and function at baseline and 1 month after TRE. We used the Wilcoxon signed rank test to compare continuous variables and McNemar's test to compare categorical variables. Results: There were 41 patients in our cohort. Median age at PH diagnosis was 98 days (IQR, 41–164). Etiologies included bronchopulmonary dysplasia (*n* = 25, 61%), congenital diaphragmatic hernia (*n* = 5, 12%), atypical alveolar capillary dysplasia (*n* = 2, 5%), and other lung diseases (*n* = 9, 22%). Median age at TRE initiation was 171 days (IQR, 123–245). After 1 month, RV function improved by mean tricuspid annular plane systolic excursion Z‐score (−2.71 vs −1.34, *p* = 0.01), fractional area change (32.9% vs 39.2%, *p* = 0.02), global longitudinal strain (−14.3% vs −20.9%, *p* < 0.01), tricuspid annulus Z‐score (1.97 vs 1.49, *p* = 0.02), and proportion of patients with normal qualitative RV function (48% vs 90%, *p* < 0.01). PH severity improved, as measured by proportion with interventricular septal bowing (41% vs 16%, *p* = 0.01), moderate/severe RV hypertrophy (77% vs 39%, *p* < 0.01), and mean RV systolic pressure by TR jet (70 mmHg vs 48 mmHg, *p* < 0.01). Observed side effects within the first 48 h of TRE were hypotension (*n* = 12, 29%), for which six patients (15%) required inotropes, hypoxia (*n* = 8, 20%), and gastrointestinal upset (*n* = 1, 2%). TRE dose was reduced in four patients and discontinued in one patient. Conclusions: TRE is associated with improvement in PH severity and RV function in Group 3 PH 1 month after initiation, but early side effects may limit use.

## A012 INTRODUCING USE OF REMUNITY® PUMPS FOR INPATIENT USE IN GROWING PEDIATRIC PULMONARY HYPERTENSION PROGRAM

Fitzgerald, Dacey^1^; Garcia, Victoria^2^; Adkins, Lindsay^1^; Shipley, Brandon^1^; Moravec, Cayce^1^; Birely, Alexandra^1^; Grapsy, Jillian^1^; Griffiths, Megan^2^

^1^Children's Medical Center, Dallas, Texas, USA

^2^University of Texas Southwestern Medical Center, Dallas, Texas, USA

Background: Offering long‐term inpatient Treprostinil infusions is a challenge for new pediatric PH programs. Options for subcutaneous therapy are limited by the unavailability of CADD MS3 pumps (Smith Medical). Central lines risk infection and thrombosis. Remunity® pumps (United Therapeutics) present barriers including single‐use recommendation, limited concentrations of Treprostinil, and the need for staff training. Benefits include noninvasive infusions, pump availability, portability, and familiarizing families with devices before discharge. Commercially‐available concentrations of Treprostinil are prohibitive to allow pediatric patients to initiate Treprostinil at home. We present our experience developing a program for using Remunity® pumps for long‐term inpatient therapy. Materials and Methods: Collaborating between pulmonary hypertension, pharmacy, biomedical engineering, and nursing, we reviewed options for inpatient Treprostinil infusions: intravenous PIV/central line via hospital IV pumps, SubQ via hospital syringe pumps, SubQ via Remunity® pumps. Four Remunity® pumps were initially purchased by the hospital, with subsequent purchase of six more. PH program coordinator, pharmacy, and nursing leadership developed a comprehensive training program for bedside staff including formal nursing competency and hands‐on training. Bedside pump kits with practical guides were developed based on staff feedback. Bedside staff reports satisfaction with the pumps, preferring them over syringe pumps due to portability during activity (e.g., cares, PT/OT). Innovations: Inpatient pharmacy protocolized new dilutions for use in Remunity® pumps, allowing the smallest patients to successfully titrate Treprostinil without a need for IV access. Pharmacy and EPIC teams built custom orders with dilution information visible to pharmacy and nursing. Drug dilution/stability data provided by United Therapeutics. Workflow considerations: Pumps are maintained per usual hospital equipment policies for multiple uses. Superusers support other nursing staff in daily management of pumps and sites. Formal hospital competency consists of an assigned pre‐test, attending an in‐service by the PH team, demonstration and simulation of site placement and cassette change with check‐off by superuser, and formal posttest to assess for competency. Quarterly in‐services scheduled for continued competency and education of new staff. Two safety events occurred without patient harm: misplaced remote, site pulled out with the loss of pump during diaper change. Results: Being able to offer SubQ Treprostinil therapy inpatient has resulted in 686 fewer central line days, decreasing infection and thrombotic risk, allowing patients increased mobility while granting patients, parents, and caregivers time to learn site placement and pump operation leading to successful management at home after discharge. Fifteen certified nursing superusers were designated with competency in managing SubQ sites, routine management of pump alarms, and supporting other nurses. Conclusions: Using SubQ Remunity® pumps in inpatient has been fundamental to a growing PH program. Utilizing a collaborative approach, we addressed the barriers to inpatient pediatric subcutaneous Treprostinil, including pump availability, concentration, and dose concerns as well as staff education. Collaboration between hospital administration, pharmacy, quality/safety, education, and clinical departments, as well as conducting targeted education for staff, can lead to the successful implementation of Remunity® pumps inpatient. Acknowledgements: We would like to acknowledge Kari Roberts and Amy Kimber from United Therapeutics for training and device support, the Children's Medical Center inpatient pharmacy, the Children's Heart Center nursing staff, and the Children's Pediatric Advanced Cardiac Care (PACC) team for their cooperation and support.

## A013 REFRACTORY PULMONARY ARTERIAL HYPERTENSION IN STAGE 1 SINGLE VENTRICLE INFANT

Zoretic, SE (fellow); Griffiths, M

University of Texas Southwestern, Dallas, Texas, USA

Background: Severe pulmonary arterial hypertension (PAH) in stage 1 single ventricle infants is a serious complication known to be associated with poor outcomes and may be a barrier to further single ventricle palliation. Clinical Case: A 4‐month‐old male postnatally diagnosed with hypoplastic left heart syndrome underwent Norwood/Sano operation at 6 days complicated by Sano revision on postoperative day 1. Cardiac catheterization at 2 weeks old demonstrated severely elevated pulmonary artery (PA) pressure with a transpulmonary gradient of 23 mmHg and pulmonary vascular resistance (PVR) of 13 indexed Wood units despite inhaled nitric oxide (iNO), epoprostenol, and 100% oxygen. He was electively cannulated onto veno‐arterial ECMO and initiated on Sildenafil. He was successfully decannulated and tolerated weaning off Sildenafil 1 month later (2 months old). At 4 months of age, he had refractory hypoxemia with repeat catheterization showing Sano shunt narrowing, mean PA pressures of 26–28 mmHg with an end‐diastolic pressure of 9 mmHg on iNO and 100% oxygen. He was started on inhaled epoprostenol for recurrent pulmonary hypertensive crises, followed by transfer to our institution for further management. On arrival, he had supra‐physiologic blood pressures on epinephrine, saturations in the 80's on 100% oxygen, 40 parts per million iNO, and 100 ng/kg/min inhaled epoprostenol. He was slowly transitioned to intravenous epoprostenol with slow decreases in inhaled agents. A repeat catheterization showed substantial improvement in PA pressures and PVR, however, with worsening diastolic dysfunction requiring milrinone intermittently. He was unable to wean off epoprostenol and was transitioned to subcutaneous treprostinil followed by Riociguat. After a period of convalescence, he is tolerating a slow wean of treprostinil and no longer requires intensive care. Conclusions: Single ventricle patients with severe PAH pose significant challenges for medical and surgical management. Targeted PH therapy can palliate these patients during recovery and rehabilitation while the next stages are planned.

## A014 CIRCULATING LEVELS OF BONE MORPHOGENIC PROTEIN 7 AND CARBOXYL TERMINUS OF HSC70‐INTERACTING PROTEIN ARE ELEVATED IN PEDIATRIC PULMONARY HYPERTENSION COMPARED TO CONTROLS

Kirkpatrick EC^1,2^, Afolayan AJ^1,2^, Handler S^1,2^, Liegl M^2^, Pan A^2^

^1^Children's Wisconsin, Milwaukee, Wisconsin

^2^Medical College of Wisconsin, Milwaukee, Wisconsin, USA

Background: The transforming growth factor beta (TGF‐β) consists of ligands and receptors that regulate arterial endothelial and smooth muscle cell homeostasis by balancing ligands and modulators of the bone morphogenic protein (BMP) and TGF‐β receptor pathways. Imbalance has been linked to the pathological changes in pulmonary hypertension (PH). These circulating protein levels may provide diagnostic and therapeutic targets for PH. This pilot study's purpose was to determine differences in these protein levels between PH patients and controls. Materials and Methods: Ten PH and 20 control subjects were prospectively enrolled. Serum was drawn from the pulmonary artery during catheterization, and circulating BMP 2, 4, 6, 7, 9, 10, activin A, TGF‐β1, CHIP, NT Pro BNP, and CRP were measured by ELISA. Subjects were only studied once. Subjects were clinically stable without medication changes. Control subjects had elective closure of a PDA or ASD and were otherwise healthy. Analyses were made between protein levels, PH status, and catheterization measurements using the Fisher's exact test, the Mann–Whitney test, and Pearson and Spearman correlations as appropriate. ROC analysis was performed with BMP7 and CHIP levels. Results: Subjects were 2–17 years with 70% group I and 30% group 3 PH. Only BMP7 and CHIP levels were statistically elevated in PH patients vs controls; (BMP7 0.081(0.076–0.084) vs 0.074(0.069–0.08), *p* = 0.044), (CHIP 0.17(0.14–0.24) vs 0.13(0.12–0.15), *p* = 0.007) respectively. BMP7 levels had a moderate correlation with RV systolic pressure (0.431 *p* = 0.02) and PVRI (0.446 *p* = 0.013). CHIP had a moderate correlation with mean pulmonary artery pressure (0.449 *p* = 0.013) and Rp/Rs (0.419 *p* = 0.02). BMP7 OD of 0.077 had a sensitivity of 80% and specificity of 70% for PH. CHIP OD of 0.136 had a sensitivity of 90% and a specificity of 65% for PH. Conclusions: Circulating BMP7 and CHIP levels were significantly elevated in PH patients compared to controls. These could provide sensitive markers for PH to aid in diagnosis and disease monitoring.

## A015 CELLULAR COMMUNICATION NETWORK PROTEINS ARE NOVEL BIOMARKERS FOR HEMODYNAMICS AND FUNCTIONAL STATUS IN PULMONARY ARTERIAL HYPERTENSION

Schramm, JE^1^; Yang, J^1^; Griffiths, M^2^; Brandal, S^1^; Damico, R^1^; Vaidya, D^1^; Simpson, C^1^; Kolb, TM^1^; Pauciulo, M^3^; Nichols, WC^3^; Ivy, DD^4^; Austin, ED^5^; Hassoun, PM^1^; Everett, AD^1^

^1^Johns Hopkins University, Baltimore, Maryland, USA

^2^UT Southwestern Medical Center, Dallas, Texas, USA

^3^University of Cincinnati College of Medicine, Cincinnati, Ohio, USA

^4^Children's Hospital Colorado, Denver, Colorado, USA

^5^Vanderbilt University Medical Center, Nashville, Tennessee, USA

Background/Hypothesis: CCN proteins are a diverse group of cysteine‐rich secreted matricellular proteins, most of which contain four tandem modules; IGF‐binding domain, von Willebrand type C, thrombospondin type I repeat, and a C‐terminal domain (CT). These multifunctional proteins regulate fundamental biological processes of high relevance to PAH pathogenesis. As such, CCNs may serve as biomarkers to predict PAH outcomes, clinical variables, and hemodynamics better than previous markers (i.e., NT Pro‐BNP). Materials and Methods: Serum CCN 1–6 proteins were measured in 40 adults with PAH and 40 age and gender‐matched healthy controls. A Mann–Whitney U test was used to compare CCN levels in PAH and controls. The relationship between CCN levels and clinical variables (including cardiac output (CO) and pulmonary vascular resistance (PVR)), functional measures such as 6‐min walk distance (6‐MWD), and New York Heart Association Functional Class (NYHA FC)) were analyzed by Spearman's Rank Correlation. Analysis of composite outcome (death or transplant) was conducted using a Cox proportional hazard model adjusted for age and sex. Results: There was a statistically significant difference in CCN levels between PAH patients and controls in all CCN proteins (*p* < 0.02), except CCN4 and CCN5. CCN6 levels correlated with decreased CO (*p* = 0.03) and increased PVR (*p* = 0.02). CCN1 is associated with a worse functional class and worse 6‐MWD, CCN5 with worse 6‐MWD, CCN2 with worse survival, and CCN6 with lower survival and worse functional class (Table 1). Conclusions: CCNs are promising novel biomarkers in PAH in adults with CCN6, the most promising in initial study. Validation is ongoing with larger cohorts to confirm utility as a PAH clinical and outcome marker.